# Angiogenic proteins, placental weight and perinatal outcomes among pregnant women in Tanzania

**DOI:** 10.1371/journal.pone.0167716

**Published:** 2016-12-09

**Authors:** Chloe R. McDonald, Anne M. Darling, Enju Liu, Vanessa Tran, Ana Cabrera, Said Aboud, Willy Urassa, Kevin C. Kain, Wafaie W. Fawzi

**Affiliations:** 1 Department of Global Health and Population, Harvard T.H. Chan School of Public Health, Boston, MA, United States of America; 2 SAR Laboratories, Sandra Rotman Centre for Global Health, University Health Network-Toronto General Hospital, University of Toronto, Toronto, Canada; 3 Department of Nutrition, Harvard T.H. Chan School of Public Health, Boston, MA, United States of America; 4 Department of Epidemiology, Harvard T.H. Chan School of Public Health, Boston, MA, United States of America; 5 Department of Microbiology and Immunology, Muhimbili University of Health and Allied Sciences, Dar es Salaam, Tanzania; 6 Tropical Disease Unit, Division of Infectious Diseases, Department of Medicine, University of Toronto, Toronto, Canada; Duke University, UNITED STATES

## Abstract

**Introduction:**

Placental vascular development, and ultimately placental weight, is essential to healthy fetal development. Here, we examined placental weight in a cohort of Tanzanian women in association with angiogenic proteins known to regulate placental vascular development and perinatal outcomes.

**Methods:**

A total of n = 6579 women with recorded placental weight were included in this study. The relative risk of adverse perinatal outcomes (Apgar score, death, asphyxia, respiratory distress, seizures, pneumonia and sepsis) was compared between placental weight in the bottom and top 10^th^ percentiles. We quantified angiogenic mediators (Ang-1, Ang-2, VEGF, PGF and sFlt-1) in plasma samples (n = 901) collected between 12 to 27 weeks of pregnancy using ELISA and assessed the relative risk of placental weight in the bottom and top 10^th^ percentiles by protein levels in quartiles.

**Results:**

Women with Ang-2 levels in the highest quartile had an increased relative risk of placental weight in the bottom 10^th^ percentile (RR = 1.45 (1.10, 1.91), p = 0.01). Women with VEGF-A (RR = 0.73 (0.56, 0.96), p = 0.05) and PGF (RR = 0.58 (0.44, 0.72), p = 0.002) in the highest quartile had a reduced relative risk of placental weight in the bottom 10^th^ percentile. Low placental weight (in bottom 10^th^ percentile) was associated with an increased relative risk of Apgar score of <7 at 1 minute (RR = 2.31 (1.70, 3.13), p = 0.001), at 5 minutes (RR = 3.53 (2.34, 5.33), p = 0.001), neonatal death (RR = 5.02 (3.61, 7.00), p = 0.001), respiratory distress (RR = 4.80(1.71, 13.45), p = 0.001), and seizures (RR = 4.18 (1.16, 15.02), p = 0.03).

**Discussion:**

The association between low placental weight and risk of adverse perinatal outcomes in this cohort suggests that placental weight could serve as a useful indicator, providing additional insight into high-risk pregnancies and identifying neonates that may require additional monitoring and follow-up.

## Introduction

Fetal development is critically dependent on the growth, structure and function of the placenta. At term the vascular network of the placenta is over 550 km in length and 15 m^2^ in surface area [1, 2]. As the sole source of oxygen and nutrition for the fetus, disruptions in placental growth and function can have serious implications on in utero development. Major complications of pregnancy, including gestational diabetes mellitus, intrauterine growth restriction, pre-eclampsia, and preterm delivery, have been linked to placental vasculature dysfunction [1, 3–7]. Placental weight at delivery is reflective of growth in the chorionic disk, placental thickness, arborisation of the chorionic villi and total surface area for nutrient exchange with the fetus, and is therefore considered an indicator of fetal development [1, 8–10]. Disruptions to placental growth can have long-term consequences on perinatal and childhood health and have been associated with obstetric outcomes (intrauterine growth restriction and preeclampsia, maternal disease), perinatal mortality and morbidity, as well as childhood growth and development [10–16].

Recent research suggests that the pathophysiological events leading to adverse birth outcomes (preeclampsia, intrauterine growth restriction, preterm birth and low birth weight) occur early in pregnancy and are closely tied to vasculogenic and angiogenic processes in the placenta, which may be reflected in the placental weight [14, 15, 17, 18]. Vasculogenesis, the de novo formation of new vasculature, begins at the third week post-conception and continues throughout the first trimester and into the beginning of the second trimester. Once formed, all villi continuously undergo angiogenesis, including both transformation (branching and elongation of capillaries) and maturation/re-modelling of vessels, throughout gestation [1, 19, 20]. Placental vascularization is closely regulated across pregnancy by the vascular endothelial growth factor (VEGF) family of proteins and the angiopoietin-Tie2 axis [18, 21, 22]. In complicated pregnancies disruptions in the balance of pro- and anti-angiogenic factors can alter placental vascular development and consequently the weight of the placenta.

There are limited data reported on the relationship between angiogenic proteins, placental weight and perinatal outcomes in resource-constrained settings. The association between placental weight and clinical outcomes established in the literature suggests that this may be an easily assessed clinical indicator of in utero growth of the fetus, mechanisms of intrauterine growth restriction and risk of adverse perinatal outcomes. Here, we examined placental weight in a cohort of Tanzanian women in association with angiogenic proteins measured at mid-gestation and perinatal outcomes.

## Methods

### Study Population

The study cohort was nested within a larger prospective trial of multivitamin supplementation in pregnancy, conducted in Dar es Salaam, Tanzania (NCT00197548, A Trial of Multivitamins and Adverse Pregnancy Outcomes). The results and trial design were previously reported in detail [23]. Briefly, HIV negative women between 12 to 27 weeks gestation (based on last menstrual period) presenting at antenatal clinics in Dar es Salaam were invited to participate. All women, in both daily supplementation and placebo arms, were given daily doses of iron (60 mg of elemental iron) and folic acid (0.25 mg) as well as presumptive anti-malarial therapy with sulfadoxine-pyrimethamine tablets (Fansidar, Roche) at 20 and 30 weeks gestation as described [23]. Any participants with missing data on placental weight were excluded from the analysis. The parent trial enrolled a total of N = 8468 women. All women with recorded placental weight (n = 6579) were included in this study. Quantification of circulating angiogenic factors in maternal blood samples was performed in a subset of women (n = 901). Selection criteria for samples in biomarker testing were defined based on inclusion criteria in a parallel study examining preterm birth and small-for-gestational age outcomes, which are higher in primigravida. We selected pre-treatment samples (all samples were collected prior to commencement on study treatment regimen) from all primigravid participants with singleton live births, known birth outcome, recorded birth weight and placental weight in this study.

### Ethics Statement

This research received ethical approval from review boards at the Muhimbili University College of Health and Allied Sciences (Dar es Salaam, Tanzania), the Harvard School of Public Health (Boston, Massachusetts) and the University Health Network (Toronto, Canada). Signed consent forms were obtained from all participants enrolled in the multivitamin supplementation trial to permit blood collection and protein analysis of plasma samples.

### Enzyme-linked Immunosorbent Assay

All blood samples were collected (a single sample per woman) prior to assignment to treatment arms (daily micronutrient supplementation or placebo). A total of 901 samples were processed and included in this cohort. Maternal peripheral plasma samples were collected and stored in EDTA at -80°C prior to testing. Plasma samples were assessed for levels of Angiopoietin-1 (Ang-1), Angiopoietin-2 (Ang-2), Vascular Endothelial Growth Factor (VEGF-A), Soluble fms-like tyrosine kinase 1 (sFlt-1), and Placental Growth Factor (PGF) using commercially available enzyme linked immunosorbent assay (ELISA) kits (Duosets, R&D Systems, Minneapolis, MN). All sample processing and biomarker assays were performed blinded to the patient trial arm. All proteins were selected based on previous evidence supporting a well-established role in placental vasculogenesis and angiogenesis [1, 3, 19].

### Outcomes

Study nurses weighed placentas and infants, to the nearest 10 grams, immediately following delivery. All placentas were weighed with membranes and umbilical cord intact. No blood was removed from the placenta prior to weighing. Neonatal death was defined as death within the first 28 days. A study physician made clinical diagnoses of respiratory distress, asphyxia, pneumonia, sepsis, and seizures post-delivery.

### Statistical Analysis

Statistical analysis was performed using STATA v13 (StataCorp., College Station, TX), SAS v9.2 (SAS Institute Inc., Cary, NC), and GraphPad Prism v6 (GraphPad Software Inc., La Jolla, CA) software. Concentrations of proteins showed deviation from normality (Shapiro-Wilks *p* < 0.05), and therefore the natural log transformation was used. Correlations between maternal demographics and placental weight were examined using Pearson’s correlation coefficient. Log binomial regression with the log link function was used to estimate relative risks and 95% confidence intervals for the associations between proteins and extreme deciles of placental weight and between placental weight and birth outcomes. When log binomial models failed to converge, log poisson models were used instead [24]. Baseline variables associated with the primary outcome of interest, placental weight, at a *p*-value less than 0.2 were included in the multivariate models for the association between proteins and placental weight. For the association between proteins and placental weight these covariates included maternal age (continuous), marital status (yes/no), literacy (literate/illiterate), baseline gestational age (<20 weeks, 20–25 weeks, >25 weeks), and trial arm (multivitamin supplementation/placebo control). We tested for the presence of linear trends by assigning each quartile the median value and modeling this variable as a continuous variable. Multivariate models for the association between placental weight and birth outcomes were adjusted for treatment arm (multivitamin supplementation/placebo control), maternal age (continuous), sex of child (male, female), gestational age at trial enrolment (weeks), and gestational age at delivery (weeks).

## Results

Baseline characteristics of the study cohort are reported in Table 1. The study cohort consisted of 6,637 women. A total of 464 women had a placental weight in the bottom 10^th^ percentile (<400 g) and a total of 560 women had a placental weight in the top 10^th^ percentile (>600 g). Placental weight and birth weight were positively correlated (r = 0.54, P < 0.001). Placental weight was positively associated with maternal age (r = 0.10, P < 0.001), gravidity (r = 0.10, P < 0.001), and BMI at baseline (r = 0.14, P < 0.001).

**Table 1:** Baseline descriptive characteristics of the study cohort (n = 6,579)

		N (%) or Median [IQR]
Treatment group	Multivitamin	3,317 (50.42)
	Placebo	3,262 (49.58)
Parity	≤1	4,691 (71.68)
	>1	1,888 (28.32)
Maternal age		24.52 [21.52, 28.53]
Marital status	Married/cohabiting	5,527 (84.67)
	Divorced/single/widowed	1,001 (15.33)
Education (years)	0–4	612 (9.34)
	5–7	303 (4.63)
	8–11	4,710 (71.91)
	≥ 12	925 (14.12)
Body Mass Index (kg/m^2^)		23.97 [21.83, 26.71]
MUACS (cm)		26 [24, 28]
Gestational age at enrolment		21.6 (19, 24)
Hemoglobin (g/dL)		10.4 [9.4, 11.2]
Anemia at enrollment		3,887 (67.64)
Malaria) at enrollment		122 (1.89)
Sex of child	Female	3,166 (48.22)
	Male	3,400 (51.78)
Birth weight (g)		3100 [2800, 3500]
Small for gestational age		875 (14.87)
Large for gestational age		860 (14.61)
Gestational age at delivery		39.7 [38, 41]
Delivery <34 weeks		341 (5.18)
Delivery <37 weeks		1,092 (16.60)

Baseline characteristics of the women with samples processed for the protein analysis are presented in S1 Table. The inter-assay coefficient of variability (CV) for all protein assays (Ang1, Ang2, VEGF-A, PGF, and sFlt-1) was <11% and the intra-assay CV was <7%. Women with Ang-2 (RR = 1.45 (1.10, 1.91), p = 0.047) in the highest quartile at enrolment had an increased relative risk of placental weight in the bottom 10^th^ percentile, while women with VEGF-A (RR = 0.73 (0.56, 0.96), p = 0.013) and PGF (RR = 0.58 (0.44, 0.72), p = 0.002) in the highest quartile had a reduced relative risk of placental weight at or below the 10^th^ percentile (Table 2). Risk of placental weight at or above the top 10^th^ percentile was not associated with levels of angiogenic proteins at enrolment (Table 2). Levels of Ang-1 and sFlt-1 were not associated with extreme deciles of placental weight.

**Table 2:** Multivariate relative risk for placental weight according to biomarker levels by quartile

**Placental Weight <10**^**th**^ **Percentile**					
	**Q1**	**Q2**	**Q3**	**Q4**	**P-trend**
Ang1	1.00 (Ref)	1.18 (0.87, 1.61)	1.42 (1.07, 1.89)	1.20 (0.89, 1.63)	0.1855
Ang2	1.00 (Ref)	1.19 (0.89, 1.59)	0.99 (0.74, 1.35)	1.45 (1.10, 1.91)	**0.0132**
VEGF-A	1.00 (Ref)	0.89 (0.66, 1.20)	0.84 (0.65, 1.09)	0.73 (0.56, 0.96)	**0.0466**
PGF	1.00 (Ref)	0.75 (0.57, 0.98)	0.67 (0.50, 0.89)	0.58 (0.44, 0.77)	**0.0019**
sFlt-1	1.00 (Ref)	1.04 (0.80, 1.36)	0.91 (0.69, 1.21)	0.80 (0.59, 1.07)	0.0637
**Placental Weight >90**^**th**^ **Percentile**					
Ang1	1.00 (Ref)	1.14 (0.66, 2.11)	1.36 (0.75, 2.47)	1.22 (0.66, 2.27)	0.5026
Ang2	1.00 (Ref)	0.96 (0.54, 1.72)	0.97 (0.54, 1.72)	0.88 (0.48, 1.62)	0.6837
VEGF-A	1.00 (Ref)	1.09 (0.56, 2.19)	1.07 (0.60, 1.90)	1.29 (0.76, 2.21)	0.3422
PGF	1.00 (Ref)	1.30 (0.66, 2.56)	0.18 (0.58, 2.41)	1.62 (0.85, 3.14)	0.1437
sFlt-1	1.00 (Ref)	0.82 (0.46, 1.45)	0.70 (0.38, 1.27)	0.70 (0.38, 1.28)	0.3322

Multivariate models adjusted for maternal age, literacy (yes/no), marital status (yes/no), baseline gestational age (weeks), and trial arm (multivitamin supplementation/placebo). P-trend (p-value) for linear trend test calculated with median biomarker in each quartile as a continuous variable.

**Fig 1:**
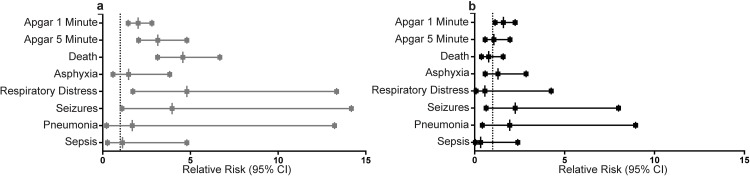
Low placental weight is associated with an increased risk of adverse perinatal outcomes. Forest plots of adjusted relative risk for perinatal outcomes with placental weight in (a) the bottom tenth percentile and (b) the top tenth percentile. Forest plots depict adjusted relative risk and 95% confidence interval. Multivariate models adjusted for treatment arm (placebo, multivitamin), maternal age (years), sex of child (male, female), gestational age at trial enrolment (weeks), and gestational age at delivery (week).

Placental weight below the 10^th^ percentile was associated with an increased risk of adverse perinatal outcomes (Fig 1). Low placental weight was associated with an increased relative risk of Apgar score of <7 at 1 minute (RR = 2.31 (1.70, 3.13), p = 0.001), at 5 minutes (RR = 3.53 (2.34, 5.33), p = 0.001), neonatal death (RR = 5.02 (3.61, 7.00), p = 0.001), respiratory distress (RR = 4.80(1.71, 13.45), p = 0.001), and seizures (RR = 4.18 (1.16, 15.02), p = 0.03) in multivariate models including treatment arm, maternal age, sex of the child, gestational age at study enrolment and gestational age at delivery (Table 3). High placental weight (> top 10^th^ percentile) was associated with an increased risk of an Apgar score <7 at 1 minute post-delivery (RR = 1.54 (1.08, 2.18), p = 0.02), but was not associated with an increased risk of any other adverse perinatal outcomes examined as part of this study (Table 3).

**Table 3:** Relative risk of adverse perinatal outcomes dependent on placental weight in the bottom and top 10^th^ percentiles

		Placental Weight <10^th^ Percentile				Placental Weight >90^th^ Percentile			
		Univariate		Multivariate		Univariate		Multivariate	
	n/N	RR (95% CI)	P-value	RR (95% CI)	P-Value	RR (95% CI)	P-value	RR (95% CI)	P-Value
Apgar <7 at 1 min	308	**2.44 (1.82, 3.27)**	**0.001**	**2.31 (1.70, 3.13)**	**0.001**	**1.48 (1.05, 2.10)**	**0.02**	**1.54 (1.08, 2.18)**	**0.02**
Apgar <7 at 5 min	136	**3.88 (2.63, 5.72)**	**0.001**	**3.53 (2.34, 5.33)**	**0.001**	1.17 (0.65, 2.01)	0.60	1.17 (0.64, 2.17)	0.61
Neonatal Death	171	**6.47 (4.77, 8.79)**	**0.001**	**5.02 (3.61, 7.00)**	**0.001**	0.67 (0.33, 1.30)	0.24	0.81 (0.42, 1.58)	0.54
Asphyxia	56	1.60 (0.65, 3.98)	0.31	1.66 (0.66, 4.19)	0.28	0.93 (0.34, 2.57)	0.89	0.93 (0.33, 2.56)	0.88
Respiratory Distress	20	**6.98 (2.70, 18.00)**	**0.001**	**4.80 (1.71, 13.45)**	**0.003**	0.63 (0.09, 4.76)	0.66	0.81 (0.11, 6.05)	0.84
Seizures	16	**3.76 (1.08, 13.14)**	**0.04**	**4.18 (1.16, 15.02)**	**0.03**	2.80 (0.80, 9.77)	0.11	2.77 (0.78, 9.87)	0.12
Pneumonia	12	1.48 (0.19, 11.46)	0.70	1.83 (0.24, 14.38)	0.62	2.41 (0.53, 10.95)	0.32	2.31 (0.50, 10.6)	0.28
Sepsis	33	1.13 (0.27, 4.69)	0.87	0.42 (0.06, 3.07)	0.39	0.40 (0.05, 2.96)	0.37	0.43 (0.06, 3.20)	0.41

Relative risk (RR) and 95% confidence interval (CI) for perinatal outcomes with placental weight in the bottom and top 10^th^ percentiles. Multivariate model includes treatment arm (placebo, multivitamin), maternal age (years), sex of child (male, female), gestational age at trial enrolment (weeks) and gestational age at delivery (weeks).

## Discussion

We examined placental weight in association with angiogenic mediators and obstetric outcomes in a cohort of women in Dar es Salaam, Tanzania. Low placental weight was associated with altered levels of angiogenic factors at mid-pregnancy as well as an increased risk of adverse perinatal outcomes. We observed that higher levels of Ang-2 at mid-gestation were associated with a higher relative risk of placental weight in the bottom 10^th^ percentile, while higher levels of VEGF-A and PGF were associated with a lower relative risk of placental weight in the bottom 10^th^ percentile. Placental weight in the bottom 10^th^ percentile was associated with increased risk of an Apgar score <7, respiratory distress, seizure, and neonatal death.

Our findings suggest that higher levels of pro-angiogenic VEGF-A and PGF at mid-gestation (12–27 weeks) were associated with a higher placental weight. These findings are consistent with abundant evidence in the literature indicating that VEGF-A and PGF mediate vessel growth and remodelling at mid-gestation [1, 17, 21]. Early in gestation, VEGF-A regulates the differentiation of mesenchymal cells into hemangioblastic cells in the villous core, as well as subsequent proliferation, migration and sprouting [25, 26]. VEGF-A acts primarily on VEGFR-1 (Flt-1) and VEGFR-2 (Flk-1), which are expressed on the endothelium [27]. VEGFR-1 can be alternatively spliced generating the soluble Flt-1 receptor (sFlt-1), which can bind circulating VEGF-A, removing it from circulation. Placental growth factor (PGF) binds the Flt-1 receptor and appears to play a critical role in angiogenesis across gestation [18, 25, 26, 28]. Reduced circulating levels of PGF in pregnancy have been associated with placental insufficiency, fetal growth restriction and subsequent late-onset small-for-gestational age outcomes [29, 30]. The angiopoietins, Ang-1 and Ang-2, act in a context dependent manner with VEGF-A. Ang-1 and Ang-2 generally act as antagonists, competitively binding the Tie-2 receptor. Binding of Ang-1 induces vascular maturation, whereas binding of Ang-2 causes destabilization in the microvascular network and promotes angiogenesis [18, 28]. Since it is problematic to quantify local levels of vasculogenic and angiogenic factors in the placenta, we used maternal peripheral levels as a proxy to placental-derived proteins. Our results suggest that higher circulating levels of pro-angiogenic VEGF-A and PGF at mid-pregnancy promote placental growth and are therefore associated with a reduced risk of low placental weight. In this cohort, high Ang-2 was associated with an increased relative risk of placental weight in the bottom 10^th^ percentile. Levels of Ang-2 typically decline across gestation [3]. Therefore elevated Ang-2-associated lower placental weight may reflect a dysregulation in Ang-2 levels at mid-pregnancy that precede the observed reduction in placental weight at delivery. For example, compensatory mechanisms could lead to an increase in levels of pro-angiogenic Ang-2, in pregnancies with low placental weight, in an attempt to increase vascularization in the placenta. These results suggest that in this cohort the molecular events leading to disruptions in placental development and ultimately changes in the placental weight may occur at earlier time-points in pregnancy. Early alterations in angiogenic factors may be a risk factor for low placental weight and adverse perinatal outcomes. Future studies examining angiogenic factors at multiple time points across gestation and at delivery will be required to better characterize changes in these proteins in association with placental weight and perinatal outcome.

We observed that low placental weight at delivery was associated with an increased risk of adverse perinatal outcomes including low Apgar score, respiratory distress, seizures, and neonatal death. We also observed an increased risk of low Apgar score, at 1 minute but not at 5 minutes, associated with high placental weight (> top 10^th^ percentile). We hypothesize that low placental weight, as a result of low levels of pro-angiogenic factors at mid-pregnancy, leads to inadequate nutrient support of the developing fetus and ultimately an increased risk of adverse perinatal outcomes as a result of altered in utero development. In contrast to our observations, a previous study conducted in Canada (Montreal, Quebec) reported an association between high placental weight and low Apgar, respiratory distress, seizures and neonatal death [10]. The disparity in our results may be a result of differences in the population demographics (maternal age, BMI, gravidity) of pregnant women in low versus high resource settings. While we did observe an association between high placental weight and low Apgar score at 1 minute, the absence of this association at 5 minutes or an association with any other perinatal outcomes examined in this study, doesn’t provide evidence of a strong association between high placental weight and adverse perinatal outcomes in this cohort. Several authors have proposed that high placental weight is a compensatory mechanism occurring in pregnancies with inadequate fetal growth [10, 13, 31]. Poor nutrition and high infectious disease burden in resource-constrained settings like Tanzania may mean that pregnant women in sub-Saharan Africa have other processes contributing to placental vascular development, placental insufficiency and adverse birth outcomes [23, 32]. Moreover they may lack sufficient nutritional intake to undergo the same degree of compensatory mechanisms hypothesized to account for high placental weight.

Strengths of this study include its prospective design and access to a large cohort of women with well-categorized demographic and clinical data that allows us to control for covariates known to contribute to placental weight. Limitations include the nested and observational nature of the study design. Although we set p < 0.05 as the cut off for significance in this study, type I error rates may be inflated as a result of performing multiple tests. Further limitations include the use of gestational dating by last menstrual period (LMP) at the time of enrolment in the parent clinical trial. Access to gold-standard ultrasound technology for gestational dating is limited in most resource-constrained settings necessitating the use of LMP. While we recognize that this study would benefit from the use of first trimester ultrasound, this technology was unavailable at the time of the parent study. Future studies will need to prospectively examine placental weight in relation to prevalent pregnancy complications such as, gestational diabetes, malaria in pregnancy (confirmed by histology and PCR), gestational diabetes and preeclampsia, which may impact placental weight. It is possible that this cohort had a selection bias for mother-baby dyads with recorded placental weight. Home delivery is common in this region and may explain why 23% of deliveries in the parent trial had no recorded placental weight. At home deliveries may be accompanied by higher rates of adverse outcomes, which could be a consequence of changes in placental development and placental weight. Several factors are capable of disrupting placental vasculogenesis and angiogenesis including maternal nutritional status. In this cohort of women high rates of malnutrition could also impact placental and fetal development. Multivitamin treatment in the parent trial was associated with a reduce risk of SGA and was included as a covariate in our analysis [23].

Previous research has associated maternal smoking, placental weight and changes in the placental-fetal weight ratio [31, 33]. Due to extremely low rates of smoking in this cohort (n = 32, 0.48%) we did not include smoking in the multivariate model and were unable to assess the impact of smoking on placental weight. Malaria in pregnancy also contributes to obstetric outcomes in endemic regions [34, 35]. Rates of malaria in pregnancy were also low in this cohort (n = 130, 2.05% by RDT), which precluded an examination of the impact of malaria on placental weight. Women enrolled in the parent trial lived in mostly urban areas and received presumptive anti-malarial therapy at 20 and 30 weeks gestation, which may account for the low rates of malaria in pregnancy at delivery. All women enrolled in the parent trial received iron supplementation as part of their routine antenatal care. Iron supplementation in combination with high rates of anemia at enrolment (53% of women in this cohort had baseline haemoglobin levels below 10.5 g/dL) complicated an examination of an association between maternal anemia and placental weight in this cohort.

The results of this study suggest that dysregulated levels of angiogenic factors at mid-pregnancy, including low circulating levels of pro-angiogenic VEGF and PGF, are associated with low placental weight at delivery. Low placental weight was associated with an increased risk of multiple adverse perinatal outcomes including neonatal death. We propose that placental weight, as an easily assessed indicator in low resource settings, could have clinical utility in risk-stratifying perinatal outcome. Moreover, evaluation of circulating angiogenic factors at mid-pregnancy may provide insight into the growth of the placenta and therefore risk of adverse outcomes after delivery. Easily assessed, low-cost parameters that enable early recognition of high-risk neonates could improve neonatal outcomes in resource-constrained settings.

## Supporting Information

S1 Table Baseline Characteristics of the Protein Analysis Study Cohort. Values depict median [IQR] or number (%) where applicable.
(DOCX)
