# Supplementary material for: Angiogenic proteins, placental weight and perinatal outcomes among pregnant women in Tanzania
Source: PLoS One. 2016 Dec 9;11(12):e0167716. doi: 10.1371/journal.pone.0167716 (PMC5147955; doi:10.1371/journal.pone.0167716)
Supplement: S1 Table — Values depict median [IQR] or number (%) where applicable. (DOCX) [file pone.0167716.s001.docx]

S1 Table: Baseline Characteristics of the Protein Analysis Study Cohort

| **Variable** |  | **n = 901** |
| --- | --- | --- |
| **Gestational age at enrollment (weeks)** |  | 21.7 [19.0, 24.1] |
| **Gestational age at delivery (weeks)** |  | 39.9 [38.3, 41] |
| **Maternal age (years)** |  | 20.7 [19.5, 23.5] |
| **Education (years)** |  |  |
| 0-4 |  | 69 (7.6) |
| 5-7 |  | 591 (65.6) |
| 8-11 |  | 201 (22.3) |
| ≥ 12 |  | 40(4.5) |
| **Marital status** |  |  |
| Married |  | 706 (78.7) |
| Divorced/single/widowed |  | 191 (21.3) |
| **Body Mass Index (kg/m^2^)** |  |  |
| **Baseline Hemoglobin (g/dL)** |  | 10.1 [9.1, 11] |
| **Birth Weight (g)** |  | 3000 [2800, 3300] |
| **Peripheral Malaria Parasitaemia** |  |  |
| Yes |  | 7 (0.8) |
| No |  | 894 (99.2) |
| **Literacy** |  |  |
| Yes |  | 816 (90.8) |
| No |  | 82 (9.2) |
| **Frequency of meat/fish consumption** |  |  |
| < 1x per week |  | 64 (7.2) |
| ≥ 1x per week |  | 837 (92.8) |
| **Sex of Child** |  |  |
| Male |  | 450 (50.05) |
| Female |  | 449 (49.95) |
| **Preterm Birth** |  | 141 (15.65) |
| **Small for Gestational Age** |  | 127 (14.32) |
| **Low Birth Weight** |  | 58 (6.44) |

Median [IQR] or number (%) where applicable
